# ADAR Enzyme and miRNA Story: A Nucleotide that Can Make the Difference

**DOI:** 10.3390/ijms141122796

**Published:** 2013-11-19

**Authors:** Sara Tomaselli, Barbara Bonamassa, Anna Alisi, Valerio Nobili, Franco Locatelli, Angela Gallo

**Affiliations:** 1 Laboratory of RNA Editing, Onco-haematology Department, Bambino Gesù Children’s Hospital, IRCCS, Piazza S. Onofrio 4, Rome 00165, Italy; E-Mails: sara.tomaselli@opbg.net (S.T.); barbara.bonamassa@opbg.net (B.B.); franco.locatelli@opbg.net (F.L.); 2 Hepato-Metabolic Disease Unit and Liver Research Unit, Bambino Gesù Children’s Hospital, IRCCS, Piazza S. Onofrio 4, Rome 00165, Italy; E-Mail: valerio.nobili@opbg.net; 3 Department of Pediatric Science, Università di Pavia, Strada Nuova 65, Pavia 27100, Italy

**Keywords:** microRNA, Adenosine deaminase acting on RNA (ADAR), A-to-I RNA editing, double-stranded RNA (dsRNA), non-coding sequence

## Abstract

Adenosine deaminase acting on RNA (ADAR) enzymes convert adenosine (A) to inosine (I) in double-stranded (ds) RNAs. Since Inosine is read as Guanosine, the biological consequence of ADAR enzyme activity is an A/G conversion within RNA molecules. A-to-I editing events can occur on both coding and non-coding RNAs, including microRNAs (miRNAs), which are small regulatory RNAs of ~20–23 nucleotides that regulate several cell processes by annealing to target mRNAs and inhibiting their translation. Both miRNA precursors and mature miRNAs undergo A-to-I RNA editing, affecting the miRNA maturation process and activity. ADARs can also edit 3′ UTR of mRNAs, further increasing the interplay between mRNA targets and miRNAs. In this review, we provide a general overview of the ADAR enzymes and their mechanisms of action as well as miRNA processing and function. We then review the more recent findings about the impact of ADAR-mediated activity on the miRNA pathway in terms of biogenesis, target recognition, and gene expression regulation.

## 1. Introduction

Protein-coding genes account for approximately 1% of the mammalian genome and 70%–90% of the rest can be transcribed but not translated [1]. Therefore, a large part of the human transcriptome consists of non-coding RNA sequences, (*i.e.*, UTRs, introns of protein-coding genes and non-coding RNAs, such as transfer RNAs (tRNAs), ribosomal RNAs (rRNAs), microRNAs (miRNAs), small interfering RNAs (siRNAs), piwi-interacting RNAs (piRNAs), long non-coding RNAs (lncRNAs), small nuclear RNAs (snRNAs), and small nucleolar RNAs (snoRNAs)). Both protein-coding and non-coding RNAs undergo several post-transcriptional modifications, which (partially) account for the complexity of both the transcriptome and proteome that characterizes the high level of gene regulation in higher eukaryotes [2]. Among these post-transcriptional mechanisms, RNA editing is an ubiquitous and crucial modification event that alters RNA molecules by nucleotide modification bypassing the genomic information [3,4]. There are different types of RNA editing [3], but the best characterized and frequent editing event in higher eukaryotes involves the conversion of adenosine (A) to inosine (I) in double-stranded RNA (dsRNA) regions through the action of the Adenosine Deaminase Acting on RNA (ADAR) enzymes [4–6].

Computational analysis combined with next generation sequencing (NGS) has recently been used to identify A-to-I RNA editing sites [7–10]. Reverse transcriptase recognizes Inosine as Guanosine. Therefore, an A-to-I RNA editing site can be identified when a cDNA sequence and the corresponding genomic DNA (gDNA) sequence are aligned. Surprisingly, several editing sites were found in non-coding regions of the human transcriptome (~15,000 sites, mapped in ~2000 different genes) and most of them are clustered within inversely oriented repetitive Alu elements (~90%). On the basis of this analysis, it is predicted that >85% of pre-mRNAs are possibly edited, with the vast majority being targeted in introns (~90%) and UTRs [10].

As Inosine is interpreted as Guanosine by splicing and translational machineries, A-to-I editing can change the informational content of the RNA coding molecules by altering splicing and translation processes. Moreover, Inosine has different base-pairing properties compared to Adenosine and differs from Guanosine by the loss of the N_2_ amino group (due to the ADAR deamination event), which accounts for the less strong interaction with Cytosine (two H-bound instead of three). Thus, A-to-I RNA editing has the potential to alter RNA structure by introducing bulges/mismatches or creating different base pairs (for examples, A-U base pairs can change into I:U mismatches in dsRNAs). The final picture is that ADARs can alter splicing, translation, and the dsRNA structure. It was originally thought that the main function of ADAR enzymes was their re-coding capacity. However, A-to-I editing most frequently targets non-coding sequences [9,11,12] and, recently, numerous interactions between ADARs and miRNA/siRNA pathways [13] have been discovered, which suggests a role of ADARs and A-to-I editing in RNA-mediated regulation of gene expression. In this review, we first provide a general overview of the ADAR enzymes and their mechanisms of action. We then focus on the miRNA pathway and the effects of ADAR-mediated modifications on the biogenesis and functions of miRNAs.

## 2. ADAR Family

**Figure 1:**
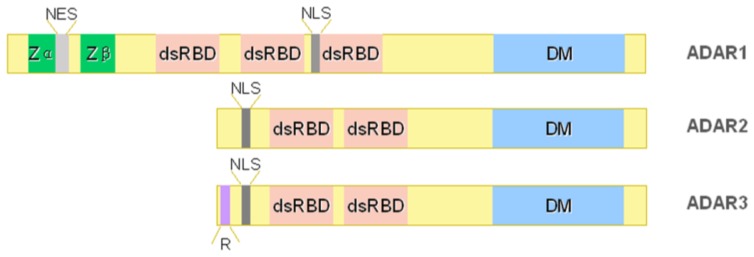
Structure of ADAR family proteins: ADAR1, ADAR2, and ADAR3. The ADAR enzymes contain a *C*-terminal conserved catalytic deaminase domain (DM), two or three dsRBDs in the *N*-terminal portion. ADAR1 full-length protein also contains a *N*-terminal Zα domain with a nuclear export signal (NES) and a Zβ domain, while ADAR3 has a R-domain. A nuclear localization signal is also indicated.

ADAR-mediated A-to-I RNA editing converts A to I by hydrolytic deamination of adenine bases. Three ADARs (ADAR1, ADAR2, and ADAR3) are present in vertebrates (Figure 1). ADARs contain a highly conserved catalytic deaminase domain (DM) at their *C*-terminal. Crystallography structure of the DM showed that the surface of this domain contains a positively-charged cleft for the binding of negatively-charged dsRNA and that it catalyses the hydrolytic deamination of Adenosine via a catalytic zinc ion [14]. Moreover, an inositol hexakisphosphate (IP6) was found buried within the enzyme core that contributes to the protein fold [14]. A nucleotide “flip-out” mechanism is necessary to force the targeted Adenosine into the catalytic pocket in the correct orientation for the deamination reaction [15].

The second key domain of all ADAR enzymes is the dsRNA-binding domain (dsRBD) at the *N*-terminus. Each dsRBD (three for ADAR1 and two for ADAR2-3) has an α-β-β-β-α topology consisting of approximately 70 amino acids, with the two α helices packing against a three-stranded anti-parallel β-sheets. Multiple dsRBDs are thought to act synergistically, which, as a consequence, increases both the affinity and specificity for dsRNA targets [16].

At the *N*-terminus, ADAR1 carries a Z-DNA binding domains (Zα plus Zβ) that suggests its localization at highly transcribed DNA sites. Moreover, as these domains can also bind Z-RNA, ADAR1 is also able to localize to underwound dsRNAs in RNA virus [17,18].

### 2.1. ADAR1

The *ADAR1* human gene is located on the long arm of chromosome 1 (1q21.3) spanning ~30Kbp [19]. The protein was discovered to exist in two isoforms of different size, *i.e.*, the interferon (IFN-α, -β, and -γ)-inducible long (ADAR1L, 150 KDa) and the constitutive short (ADAR1S, 110 KDa) isoform, which result from the use of alternative start codons and promoters [20]. While ADAR1S promoter is constitutively active, IFN can induce ADAR1L, suggesting a role in the cellular response to stress factors such as viral infections [21]. In addition to the finding of regulatory elements within the IFN-inducible ADAR1 promoter, recent studies revealed distinct tissue-specific expression features for different ADAR1 transcripts [22]. Both transcripts contain three dsRBDs but the *N*-terminus of ADAR1L has several domains that are absent in ADAR1S, including an arginine-glycine-enriched domain (RG domain) and a nuclear export signal (NES) within the Zα domain. Thus, ADAR1L is found both in the cytoplasm and nucleus since it also has a nuclear/nucleolar localization signal (NLS/NoLS) [23]. Consequently, the intracellular distribution of the various ADAR1 isoforms is determined by the export/import regulatory proteins available in a cell. On the contrary, ADAR1S localizes mainly to the nucleus since it carries only the NLS/NoLS signal. However, it has been shown that ADAR1S can also localize to the cytoplasm thanks to the cooperative action of all three dsRBDs, with dsRNAs able to interact with exportin-5 [24].

The small ubiquitin-like modifier 1 (SUMO-1) binds ADAR1 at lysine 418, decreasing the editing activity of the enzyme [25]. ADAR enzymes can form homo- and hetero-dimers and dimerization is essential for their editing activity [26,27]. Several studies have shown that ADAR1 (and ADAR2) can work as homodimer, whereas other investigations have demonstrated that also heterodimers can be formed, which may be necessary for the ADARs to act as active deaminases [27–29]. Other ADAR-interacting proteins include the nuclear factor 90 (NF90) proteins [30], the protein-kinase RNA-activated protein (PKR) [31], the adenovirus-associated (VAI) RNA [32], and the Vaccinia virus E3L protein [33].

### 2.2. ADAR2

The *ADAR2* human gene is located on the long arm of chromosome 21 (21q22.3), spanning ~153 Kbp [34]. The promoter that directs ADAR2 expression has not been functionally characterized, although a putative promoter region upstream of a newly identified exon was described for both the human and the mouse *Adar2* gene [35]. This promoter includes a TATA box sequence and the consensus binding sites for the Nuclear factor kappa-light-chain-enhancer of activated B cells (Nf-κB) and for the Specificity Protein 1 (SP1) [35]. While it has to be established whether ADAR2 possesses multiple promoters to produce multiple transcripts like ADAR1, the regulatory mechanism(s) driving the transcriptional control of ADAR2 in a tissue- and cell type-specific fashion have been partially unveiled. Indeed, it was shown that cAMP response element-binding (CREB) can indirectly induce ADAR2 expression [36]. More recently, Yang *et al.* [37] demonstrated that JNK1 serves as a crucial component in mediating glucose-responsive up-regulation of ADAR2 expression in pancreatic β-cells, suggesting that the JNK1 pathway may be functionally linked to the nutrient-sensing actions of ADAR2-mediated RNA editing in professional secretory cells.

ADAR2 *N*-terminus has an arginine-enriched domain (R-domain) (similar to that identified in the ADAR3 protein, Figure 1) that contains a NLS [35,38], while an extra NLS is located before the first dsRBD [35,39]. Consequently, ADAR2 localizes into the cell nuclei thanks to the action of importin α1, α4, and α5 [39].

ADAR2 can form homodimers and heterodimers with ADAR1 [27–29]. ADAR2 dimerization seems to be essential for editing activity, although it is not clear whether the interaction is or not dsRNA-mediated [27,40,41].

### 2.3. ADAR3

The *ADAR3* human gene is located on the short arm of chromosome 10 (10p15) in proximity of the telomere [42]. Although ADAR3 has conserved all the key catalytic residues of the ADAR family members, no deaminase activity has been found for this enzyme so far [43]. All the editing sites have been, thus, attributed to ADAR1 and 2 activity. ADAR3 protein carries two dsRBDs and, additionally, an R-domain that binds single-stranded RNAs (ssRNAs) [43,44], suggesting that both ss- and dsRNAs can be bound by the enzyme. ADAR3 is localized in the nucleus of the cell and interacts with the importin α1 through the R-motif [35].

Differently from the ubiquitously expressed ADAR1 and 2, ADAR3 is expressed at detectable levels only in certain post-mitotic cells in the central nervous system (CNS) [43]. Furthermore, ADAR3 remains in the monomeric form, which may explain the lack of editing activity, at least in part [28]. Thus, ADAR3 function is unknown so far, although its ability to bind both ss- and dsRNAs would suggest a regulatory activity over ADAR1 and 2. Indeed, ADAR3 can compete for dsRNA substrates preventing the binding of the other ADAR enzymes [43,45].

## 3. ADAR Substrates

Any dsRNAs of ≥20 bp can be an ADAR substrate [6]. ADAR substrates were originally identified by chance, comparing cDNAs to their genomic counterparts and finding editing events as a mixture of A/G instead of A only. Different editing sites have been identified over the years, particularly in transcripts coding for proteins expressed in the CNS [4,5,46], *i.e.*, those coding for subunits of the glutamate receptor super-family GluR, the serotonin 5-hydroxytryptamine 2C (5-HT2C)-receptor, the potassium voltage-gated channel (Kv1.1), and the a3 subunit of the γ-aminobutyric acid (GABAA) receptor. These editing events have a major impact on protein properties.

More recently, bio-computational studies and innovative sequencing techniques have demonstrated that A-to-I RNA editing mainly affects non-coding RNAs [6]. Importantly, the majority of editing events occur in introns and 5′–3′ UTRs enriched with Alu repeat-mediated dsRNAs. Recently, a database collecting the identified (validated or not) editing RNAs has become available (http://darned.ucc.ie) [47].

ADAR-mediated editing levels range from 2% to 100% [5,13,46], depending on cell and tissue type [48] as well as developmental stages [49]. How ADAR chooses the target adenosine is still not completely clear. ADARs show slight sequence preferences [50]. However, dsRNA length and structure seem to play an important role. For example, dsRNAs of 15–40 bp are edited selectively at very few sites, whereas those longer than 50 bp are extensively or non-selectively deaminated (with 50%–60% of adenosines being edited) [51]. Similarly, selective deamination is also observed in dsRNAs with bulges, loops, and mismatches [52]. It has been suggested that ADAR substrate specificity may also depend on editor modulators (such as snoRNAs) [53] and on the different dsRBD number and spacing of ADAR proteins that allow discrimination between dsRNA structures and stabilities. While the importance of site-specific editing (within coding sequence genes or microRNAs) has been explored and was found to affect the final protein or miRNA maturation/targeting, the role of the non-specific/promiscuous editing (within non-coding RNA portions such as introns and 5′–3′ UTRs) is still poorly understood. However, recent studies would point out their involvement in modulation of gene expression, which may occur by changing the splicing enhancers/silencers recognition sites [54–57], by perturbing/inducing the binding of RBPs for RNA nuclear localization/retention [58] or inducing inosine-specific degradation (Tudor-SN nuclease) [59].

## 4. miRNA World Machinery Overview

**Figure 2:**
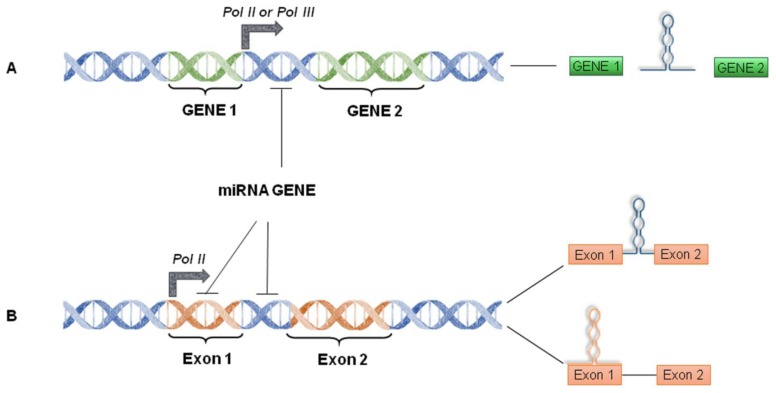
Schematic diagram of the miRNA genes. (**A**) Monocistronic intergenic miRNA gene; (**B**) Monocistronic exonic/intronic miRNA gene.

miRNAs are short (~20–23 nucleotides) ssRNAs that regulate, at post-transcriptional level, several genes playing crucial roles in various cellular processes such as cell cycle, apoptosis, differentiation and, when deregulated, neoplastic transformation [60]. Mammalian miRNA genes (in cluster or as single unit) are located either in introns/exons of protein-coding genes, in non-coding genes, or in intra-genic regions of the genome [61–63] (Figure 2). Intronic/exonic miRNAs are often transcribed by the RNA polymerase (Pol) II and co-expressed with their host gene, while intergenic miRNAs are independently transcribed by either RNA Pol II or III [64,65]. Usually, miRNA promoters located in the inter-genic or non-coding regions of the genome are regulated by transcriptional or epigenetic factors like protein-coding genes [66].

Each miRNA may regulate several mRNAs post-transcriptionally, while a single mRNA can be targeted by several miRNAs via base-pairing to the mRNA 3′ UTRs [67,68].

The conventional theory assumes that the “seed sequence” (~6–8 nucleotides in length) at miRNA 5′ end is crucial for target specificity and mediates its binding to 3′ UTRs of target mRNAs, causing their translational repression or degradation [69]. However, recent studies suggest that miRNAs can exert their action over specific targets using alternative mechanisms, including the binding to specific proteins or to non-coding RNAs [70,71]. The biogenesis and processing of miRNAs occur in the nucleus/cytoplasm due to the action of multiple proteins. Some of these have a well-known role(s) in miRNA processing, including Drosha, exportin 5, Argonaute (Ago), and Dicer, while others have partially been explored such as ADAR1 [72].

### 4.1. miRNA Biogenesis and Processing into the Nucleus

**Figure 3:**
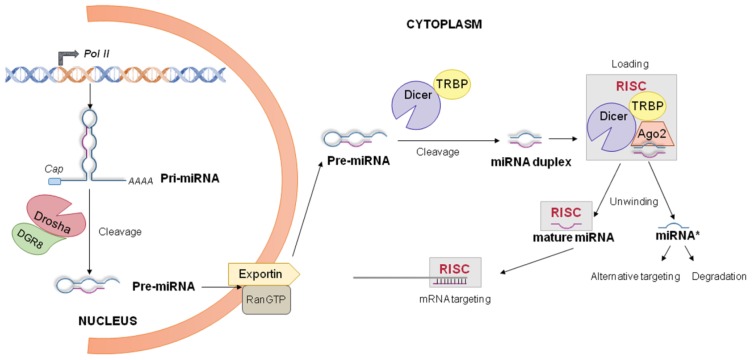
miRNA biogenesis and processing. Canonical biogenesis of pri-miRNA transcription is mediated by Pol II. Next, the microprocessor complex composed of Drosha and DGCR8 mediates the nuclear cleavage of pri-miRNA into pre-miRNA. The nuclear export of pre-miRNA is subsequently mediated by exportin-5/Ran-GTP61. Cytoplasmic pre-miRNA is processed by Dicer into a duplex microRNA. The next step is the unwinding of the duplex into a mature ~22 nucleotide miRNA and a miRNA* by the RISC complex. The mature miRNA is generally conveyed by the RISC on the targeted mRNA, whilst miRNA* can be degraded or alternatively perform a different targeting.

The early step of miRNA biogenesis in the nucleus is the transcription of a miRNA precursor (Figure 3). Mature miRNAs are generated from long, hairpin-shaped primary transcripts (pri-miRNA) that are usually several thousand nucleotides long [66]. After transcription, pri-miRNAs undergo multiple steps of processing into the nucleus. Conventional nuclear processing of pri-miRNAs happen due to their cleavage by a large microprocessor complex (650 kDa in humans) consisting of the RNase III enzyme Drosha and the DiGeorge syndrome Critical Region gene 8 (DGCR8) protein [73,74]. Specifically, Drosha, a nuclear protein of 130–160 kDa, cuts the 5′ and 3′ ends of the pri-miRNA molecule with its RNase domain, giving a short hairpin of 60–70 nucleotides long (pre-miRNA) [66]. Although DGCR8-Drosha microprocessor is involved in the cropping of many miRNAs, Drosha may also form larger complexes with other proteins (e.g., RNA helicases, dsRNA binding proteins, heterogeneous nuclear ribonucleoproteins, *etc*.) to regulate the processing of specific pri-miRNAs [75]. A recent study provides evidence that certain mature miRNAs combined with Ago proteins may re-enter the nucleus and inhibit the pri-miRNA processing [76].

Following the nuclear processing, pre-miRNAs are exported to the cytoplasm by an energy-dependent mechanism involving the exportin-5/Ran-GTP61 complex. Exportin-5 binds pre-miRNA molecules and Ran-GTP61, which catalyses GTP hydrolysis and the consequent release of pre-miRNA short precursors into the cytoplasm. Interestingly, Exportin-5 also hampers pre-miRNA nuclear accumulation, protecting them from a potential nuclear digestion and retention [77,78]. In addition to the nuclear-to-cytoplasm pre-miRNA flux, the presence of functional mature miRNAs into the nucleus suggests a retrograde transport regulated by other carriers such as Importin 8 [79].

### 4.2. miRNA Processing into the Cytoplasm

Once exported from the nucleus, the cytoplasmic pre-miRNA duplex is further processed by Dicer and other accessory proteins, including the transactivation response RNA binding protein (TRBP), the protein activator of the dsRNA-dependent protein kinase (PACT), and the Ago proteins (Figure 3). Together they form the RNA-induced silencing complex (RISC) [80–83].

For miRNAs displaying a high degree of complementarity along the hairpin stem, a preliminary Ago 2-dependent cleavage is required before Dicer action. This Ago 2 slicer activity generates a nicked hairpin, producing a precursor miRNA or ac-pre-miRNA that is further processed by Dicer [84]. Dicer typically cleaves pre-miRNA duplexes near the terminal loop, releasing a small RNA duplex of ~22 nucleotides [66].

After Dicer-mediated cleavage, the small RNA duplex is loaded onto an Ago protein (Ago 1–4 in mammals) of the RISC to generate the microRNA containing ribonucleoprotein complex, *i.e.*, miRNP or miRISC. Usually one single-strand (named guide) of the duplex (which is complementary to the target mRNA) is charged on Ago 2 as a mature miRNA, while the other strand of the duplex (named passenger or miRNA*) is usually degraded. miRNA guide (or in some cases miRNA* [85,86]) is selected to associate with Ago proteins by their thermodynamic stability [87]. There are at least two other hypotheses to explain duplex unwinding into guide and passenger strand. Dicer could cleave the miRNA*, releasing the miRNA guide that is subsequently captured by Ago 2. Alternatively, the miRNA* of a loaded duplex could be cleaved by the slicer activity of Ago 2, which simultaneously retains the miRNA guide. The activated RISC can bind the target mRNA, and direct its degradation, or repress its translation [88]. However, it has been reported that in some cases, miRNAs can also up-regulate the expression of their targets [85,89].

## 5. ADAR-Dependent Effects on miRNA Pathway

As ADARs can bind to and edit any dsRNA, the discovery that these enzymes are able to modify dsRNA substrates that enter the miRNA-mediated gene silencing and RNA interference (RNAi) pathways, *i.e.*, miRNA and siRNA precursors [13], does not come as a surprise. It has been shown that mammalian pri-miRNAs undergo A-to-I RNA editing in adult brain [86,90–92]. Furthermore, NGS analysis has shown that ADARs can alter miRNA processing and sequence in *C. elegans*, mouse embryos, human and mouse brain [93–96]. Moreover, a more recent study showed that ADAR1 forms a complex with Dicer, promoting miRNA processing, RISC loading of miRNAs and silencing of target RNAs independently of its deaminase activity [72], as previously suggested [97].

**Figure 4:**
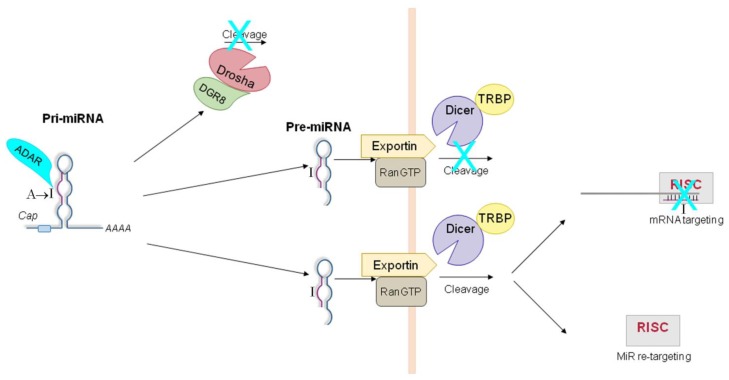
Editing-dependent effects of ADARs on miRNA pathway. miRNA precursors (pri- and pre-miRs) undergo specific A-to-I RNA editing that (i) may block their maturation process at either Drosha or Dicer step; (ii) may affect the loading of the edited miRNA to the RISC complex; (iii) may redirect the edited miRNA to a new set of target mRNAs.

In summary, several miRNA precursors (pri- and pre-miRs) undergo specific A-to-I RNA editing that may inhibit their maturation process and, thus, the production of mature miRNAs, affecting the loading of the edited miRNA to the RISC complex, or redirecting the edited miRNA to a new set of target mRNAs (Figure 4). Considering that A-to-I editing can also occur within the 3′ UTR regions of mRNAs, the picture of miRNA-ADAR interaction becomes even more complex, underlining the high level of regulation of the miRNA world.

### 5.1. ADAR-Dependent Effects on Pri-miRs

The first report of RNA editing events in a miRNA precursor dates back to almost ten years ago, when Maas and co-authors detected a low level (~5% in human brain) of A-to-I changes within the pri-miR-22 [86]. Using human cell lines (HEK293T), ectopically expressing ADAR1 or ADAR2, they found that pri-miR-22 is mainly edited by ADAR1, although the physiological role of this editing was not elucidated.

A couple of years later, Yang *et al*. confirmed that ADARs can interact with pri-miRNAs using RNA editing assays and data from *Adar1* and *Adar2* null mice [98]. Four out of the eight analyzed miRNA precursors displayed A-to-I editing *in vitro* (*i.e.*, pri-miR-142, -223, -1-1, -143), with pri-miR-142 harboring the highest editing levels. Both ADAR1S and ADAR2 are able to edit pri-miR-142 at 11 specific sites, nine of which lie within the mature miRNA sequence. Transfecting edited pri-miR-142 in HEK293 cells, the authors determined that editing at the +4 and +5 sites destroys the integrity of the stem-loop structure, inhibiting the maturation of the pri- to pre-miRs. The consequence is a reduced production of mature miR-142. Indeed, the levels of endogenous miR-142 were lower in wild-type mouse spleens than those in *Adar1* and *Adar2* null mouse spleens. However, some editing sites (such as the one at site +40) seem not to affect pri-miR-142 processing. Editing-mediated inhibition of miRNA maturation at the pri-miR step does not cause accumulation of the edited pri-miR-142, as it may be degraded by Tudor-SN, a component of the RISC complex [99], known to mediate the degradation of inosine-containing dsRNAs (IU-dsRNAs) *in vitro* [59].

The discovery that edited pri-miRs can undergo rapid degradation by Tudor-SN suggests that the amount of edited pri-miRNAs into a cell could be higher than previously hypothesized. A recent study showed that ADAR1L (the ADAR1 nucleus/cytoplasmatic shuttling isoform) and Tudor-SN co-localize in the cytoplasm within stress granules (SGs) in HeLa cells under various stress conditions [100]. The authors speculated that ADAR1 may edit target dsRNAs in the cytoplasm and the resultant IU-dsRNA may recruit Tudor-SN to form SGs during cell stress responses. However, further experiments are needed to better define the role of ADAR1 in this context and the importance of the ADAR1-mediated SG formation.

### 5.2. ADAR-Dependent Effects on Pre-miRs

Editing can also influence Dicer cleavage, which is responsible for the processing of pre- into miRNAs. This has been first demonstrated for pri-miR-151 [101]. ADAR1-dependent editing at the −1 and +3 site has been reported [90,101], which reduces the efficiency of the Dicer-TRBP activity and results in the production of unedited mature miR-151 [101]. Interestingly, editing of mouse pri-miR-151 is CNS-specific, although both ADAR1 and pri-miR-151 were found expressed in many non-brain tissues.

### 5.3. ADAR-Dependent Effects on RISC-Loading

Epstein-Barr virus (EBV) encodes 23 miRNAs that are implicated in the attenuation of host antiviral immune response and the transition from latent to lytic replication [102,103]. Among EBV miRNAs, four primary miRNAs were found to undergo site-specific A-to-I editing events [104]. The authors focused on pri-miR-BART6, which showed high editing levels at the +20 in EBV latently infected cell lines. This editing reduces the correct loading of miR-BART6-5p into the RISC complex. Remarkably, this is the first report of pri-miRNA A-to-I editing that suppresses RISC loading [104]. Editing of pri-miR-BART6 reduces the activity of mature miR-BART6, playing a crucial role in the regulation of EBV life cycle and cell immune response.

Recently, new A-to-I editing events have been reported within another EBV miRNA, *i.e.*, pri-miR-BART3. Editing was found at four sites in EBV-infected epithelial carcinoma cells and in nasopharyngeal carcinoma samples, affecting both the biogenesis and targeting of mature miR-BART3 [105].

### 5.4. ADAR-Dependent Effects on Retargeting

A specific ADAR-mediated A-to-I change has been reported in Kaposi’s sarcoma-associated herpesvirus (KSHV) transcripts [106,107]. This alteration modifies the seed sequence of the mature miR-K10, potentially affecting its target mRNAs [106,107]. ADAR1S heavily edits the K12 transcript in a specific site, as shown by *in vitro* editing assays [106]. Importantly, the authors observed that this editing event has a functional significance, playing a key role in the replication strategy of HHV-8 and in its tumorigenic potential. This was the first evidence that ADAR-mediated editing can also affect the target specificity of a mature miRNA.

Subsequently, Nishikura and colleagues demonstrated that edited mature miRNAs play a biological function *in vivo* [91]. The human pri-miR-376a1, previously showed to be edited [90], is situated in a cluster of 6 pri-miRNAs. The authors disclosed that five out of these six miRNAs are edited in human tissues (*i.e.*, pri-miR-367a1, -367a2, -367b, -368, -B2). Several adenosines within the miR-376 cluster members undergo A-to-I editing, with two positions showing the highest editing levels (nearly 100% in certain tissues), *i.e*., the +4 site, which is preferentially edited by ADAR2, and the +44 site, which is selectively edited by ADAR1. These editing events do not affect the primary transcript maturation steps. However, both +4 and +44 sites lay within the seed sequences of miR-376a* and miR-376a respectively, suggesting that the edited miRNAs could have a different target mRNA profile. In particular, the authors demonstrated that a single ADAR2-mediated base change (at the +4 site) is able to modulate the expression of phosphoribosyl pyrophosphate synthetase 1 (PRPS1), a mouse protein involved in purine metabolism and uric acid synthesis [91].

Notably, a recent work has elegantly demonstrated the existence of a tight link between miR-376a editing and human brain tumors [108]. Choudhury *et al*. found that RNA editing of miR-376 cluster is extremely reduced in human gliomas, with glioblastoma cells accumulating almost exclusively the unedited form of miR-376a*. The unedited miRNA promotes glioma cell migration and invasion, whilst the edited form inhibits these capacities *in vitro*. These effects are the consequence of a different mRNA target specificity of the edited and unedited form of the miRNA [108]. As ADAR2 is responsible for miR-376a* editing, these findings strengthen the notion that this enzyme plays a crucial role in glioma progression, as previously shown [45,109,110].

### 5.5. ADAR-Dependent Effects on Target 3′ UTRs

As most of the editing sites are also located in 3′ UTRs of human mRNAs [111], an additional interplay between ADAR activity and miRNAs is possible. Computational screening showed that RNA editing tends to avoid miRNA binding sites, with less than 10% of editing events occurring in 3′ UTR regions recognised by miRNAs [111]. However, it was also found that editing can create new miRNA target sites [111].

More recent analyses indicate that up to 20% of the editing sites in the 3′ UTR of human mRNAs may alter miRNA target sites [112], making the mRNA resistant to miRNA activity. In addition, in mouse tissues, A-to-I changes seem to be highly frequent in 3′ UTR regions, including miRNA target sites [113]. Wang *et al.* provided novel insights into the mechanism by which ADAR1 and its activity regulate miRNA-mediated modulation of target gene expression [114]. Indeed, multiple A-to-I RNA editing events (mediated by ADAR1) were found within the 3′ UTR of *ARHGAP26*, encoding the Rho GTPase activating protein 26. Furthermore, the authors revealed that both miR-30b* and miR-573 are able to target ARHGAP26, but that editing make this transcript resistant to repression mediated by these two miRNAs.

### 5.6. ADAR-Mediated Editing-Independent Effects on miRNAs

In addition to A-to-I sequence changes on miRNAs, ADARs can also act through an editing-independent mechanism by binding dsRNAs [97]. Heale *et al.* found that ADAR1 and ADAR2 editing activity can result in retargeting of human miR-376a2, as shown previously for mouse miR-376 [91]. By performing *in vitro* pri-miRNA processing assays, they also pointed out that, even in the absence of editing, ADAR2 can inhibit the processing of pri-miR-376a2 at the Drosha cleavage step [97]. Therefore, the simple binding of ADAR proteins to dsRNAs may have a range of biological roles that are still to be fully discovered.

## 6. Large-Scale Surveys

Initial low-throughput experiments followed by NGS approaches have been performed by several groups, adding new insights on the role of ADARs in the miRNA pathway.

One of the original systematic survey proposed that 6% of all human pri-miRNAs are edited [90]. The author determined that six out of 99 pri-miRNAs undergo editing (*i.e.*, pri-miR-151, -197, -223, -376a, -379, -99a) in humans. The extent of editing ranged from ~10% to 70%, depending on sites and different tissues analyzed. Most of the editing events were located in the mature miRNA seed sequence, suggesting that RNA editing may contribute to increase miRNA diversity. This paper established that ADARs edit miRNAs but did not elucidate the functional consequences of these events.

A couple of years later, a larger scale survey of 209 human pri-miRNAs showed that ~16% of them undergo A-to-I editing in human brain, with editing levels ranging from ~10% to 100% [92]. Then, for six randomly chosen edited pri-miRNAs (*i.e.*, pri-let-7g, pri-miR-33, -133a2, -197, -203, -379) it was discovered that editing alters either the Drosha or the Dicer cleavage step. It is worth noting that the processing of two pri-miRNAs (*i.e.*, pri-miR-197 and -203) was enhanced by editing. The authors also showed that some pri-miRs are preferentially edited by ADAR1 (*i.e.*, pri-miR-99b, -151, -376b, -411, -423), while others by ADAR2 (*i.e.*, pri-let-7g, pri-miR-27a, -99a, -203, -376a, -379) [92].

Recent advances in high-throughput small RNA sequencing (smRNA-Seq) have reshaped the miRNA research landscape, including RNA editing analysis. Using a novel strategy to avoid cross-mapping artefacts, de Hoon *et al*. found that editing prevalence in human mature miRNAs is extremely low in a human monocytic leukemia cell line (THP-1) [115]. Ten potential miRNA editing sites were found. However, eight of these were due to cross-mapping, one was due to a single nucleotide polymorphism, and the remaining editing site (in the mature miR-376c) was already identified [91]. Similar results were obtained by sequencing small RNAs from mouse brain [116].

Recently, Vesely and co-workers analyzed the frequency and sequence composition of miRNA pools from transgenic *Adar* null mouse embryos by NGS [93]. *Adar2* deficiency leads to a change in the expression level of specific target mRNAs when compared to wild-type embryos. In particular, the authors detected 10 edited miRNAs, four of which had been identified previously (*i.e.*, mmu-miR-378, -376b, -381, -3099) and six were novel edited miRNAs (*i.e.*, mmu-miR-1957, -467d*, -706, -1186, -3102-5p.2, -703). Some editing events were located in the seed region, opening the possibility that editing could lead to their retargeting. However, the biological consequences of the observed editing events are difficult to interpret, especially because of the low levels detected.

Using NGS followed by bioinformatics analysis, Eisenberg and co-workers found a clear A-to-I signal in mature miRNAs of human brain [94]. Overall, 19 statistically significant modification sites (mainly due to ADAR2 activity) were detected in 18 different miRNAs, confirming previously detected editing sites as well as revealing several novel ones. Most of the detected A-to-G modifications were within the miRNA seed sequence, with editing significantly changing their binding specificity. As previously reported, a relatively low editing level was found, with few exceptions (editing percentage ranging from 0.2% to 70%) [94].

## 7. Stimulative Role of ADAR1

ADAR1 has been emerging as a promoter for small non-coding RNAs. Indeed, a recent study has highlighted the important role of ADAR1 in interacting with Dicer to form heterodimers [72]. Notably, the authors established that ADAR1 uses its second dsRBD to form ADAR1/Dicer heterodimers (acting as modulator of RNAi machinery) and its third dsRBD to form ADAR1/ADAR1 homodimers (acting as an RNA editing enzyme). The ADAR1/Dicer interaction increases the rate of processing from pre- to mature miRNAs, promotes the RISC loading and, consequently, the mRNA silencing efficacy [72]. It seems that neither dsRNA-binding nor deaminase activity of ADAR1 is required for these effects. As expected, the authors found that the miRNA expression is inhibited in *Adar1* null mouse embryos, as a consequence of the lack of formation of the Dicer/ADAR1 complex with a final alteration of the target genes [72].

## 8. Conclusions

A-to-I editing is believed to be an important way of generating protein diversity by codon alteration in mRNAs. However, editing sites in some coding targets make up only a tiny fraction of all editing events, most of which are actually located in non-coding sequences such as introns, UTRs or regulatory RNAs (miRNAs and their precursors). The biological function of editing in non-coding RNA sequences remains not completely disclosed. As far as miRNAs are concerned, the general feeling about A-to-I changes is that they regulate the levels of cellular dsRNAs, which, if not kept under control, are potent triggers of gene silencing and signaling pathway. Despite this, important questions still stand. At which extent and how diffuse is the RNA editing on mature miRNAs and their precursors? Is it a developmentally regulated or a tissue specific phenomenon? In principle, editing at any level of miRNA biogenesis may have a broad influence on expression patterns. Although the evidence is still limited, a critical examination of data reported in the literature does offer some examples of miRNA down-stream activity misregulation. One more question is whether there is any correlation between edited miRNAs and human diseases. While alterations in both substrate editing and ADAR expression/activity are often reported in different pathologies [4,5,117], the effects of edited miRNA pathways on disease onset/progression still deserves further investigation. In this context, it is worth noting that Choudhury *et al.* demonstrated that a single editing event in the miR-376a* seed sequence dramatically alters the selection of its target genes and redirects its function from inhibiting to promoting glioma cell invasion [108]. Overall, these pieces of information set the stage for further investigations, either to address the aforementioned questions and, possibly, to score against ADAR/miRNA editing-linked human diseases.
